# First record of the complete chloroplast genome of the Sri Lankan endemic species, *Artocarpus nobilis* Thwaites (Moraceae)

**DOI:** 10.1080/23802359.2026.2732728

**Published:** 2026-09-30

**Authors:** Harichandra Khalingarajah, Partheepan Thavarajah, Ziqi Zhang, Zachary Rochelin, Mamadou Abdoulaye Konare, Gwendolyn Felocity Ban, Frédéric Anderson Konkobo, Lamin Manjang, Shiou Yih Lee

**Affiliations:** ^a^ Faculty of Health and Life Sciences, INTI International University, Nilai, Negeri Sembilan, Malaysia; ^b^ Department of Fisheries, Faculty of Science, University of Jaffna, Sri Lanka; ^c^ Faculty of Liberal Arts, Shinawatra University, Pathum Thani, Thailand; ^d^ College of Agriculture and Environmental Science, American University of the Caribbean, Les Cayes, Haiti; ^e^ Department of Biology, Faculty of Sciences and Techniques (FST), University of Sciences, Techniques and Technologies of Bamako (USTTB), Bamako, Mali; ^f^ School of Agriculture, Papua New Guinea University of Technology, Lae, Morobe, Papua New Guinea; ^g^ Laboratory of Biochemistry, Biotechnology, Food Technology and Nutrition (LABIOTAN), Department of Biochemistry, and Microbiology, University Joseph Ki-Zerbo, Ouagadougou, Burkina Faso; ^h^ Catholic Relief Services, Serekunda, Gambia

**Keywords:** *Artocarpus nobilis*, Ceylon breadfruit, Moraceae, genetic resources, genome structure

## Abstract

*Artocarpus nobilis* Thwaites (Moraceae), endemic to Sri Lanka and classified as Vulnerable, was subjected to complete chloroplast genome sequencing and analysis. The 161,196-bp long genome had a GC content of 36% and comprised a large single-copy region of 89,878 bp, a short single-copy region of 19,982 bp, and two inverted repeats of 25,668 bp each. It contained 131 genes, including 86 protein-coding, eight rRNA, and 37 tRNA genes. Phylogenetic analysis showed *A. nobilis* is closely related to *A. heterophyllus* and *A. integer*. These genomic data support future conservation and breeding studies.

## Introduction

*Artocarpus nobilis* Thwaites 1861, an endemic species to the wet tropical regions of southwestern Sri Lanka, is commonly known as Ceylon breadfruit or wal dhel (wal-del) by the locals (Madushani et al. 2014). The plant is a monoeceious, large evergreen tree reaching up to 25 m in height, large alternately arranged dark green leaves, producing large cylindrical syncarpous fruits (Figure 1) (Senadeera et al. 2022). The tree is valued for its edible fruit, timber, and rich phytochemicals used in Ayurveda for various ailments (Hill et al. 2025). The species is rich in bioactive compounds such as phenolics, flavonoids, antioxidants, fatty acids, and ascorbic acid, as well as exhibits notable pharmacological properties, including antioxidant, anti-inflammatory, and enzyme inhibitory activities (Hill et al. 2025).

**Figure 1.**
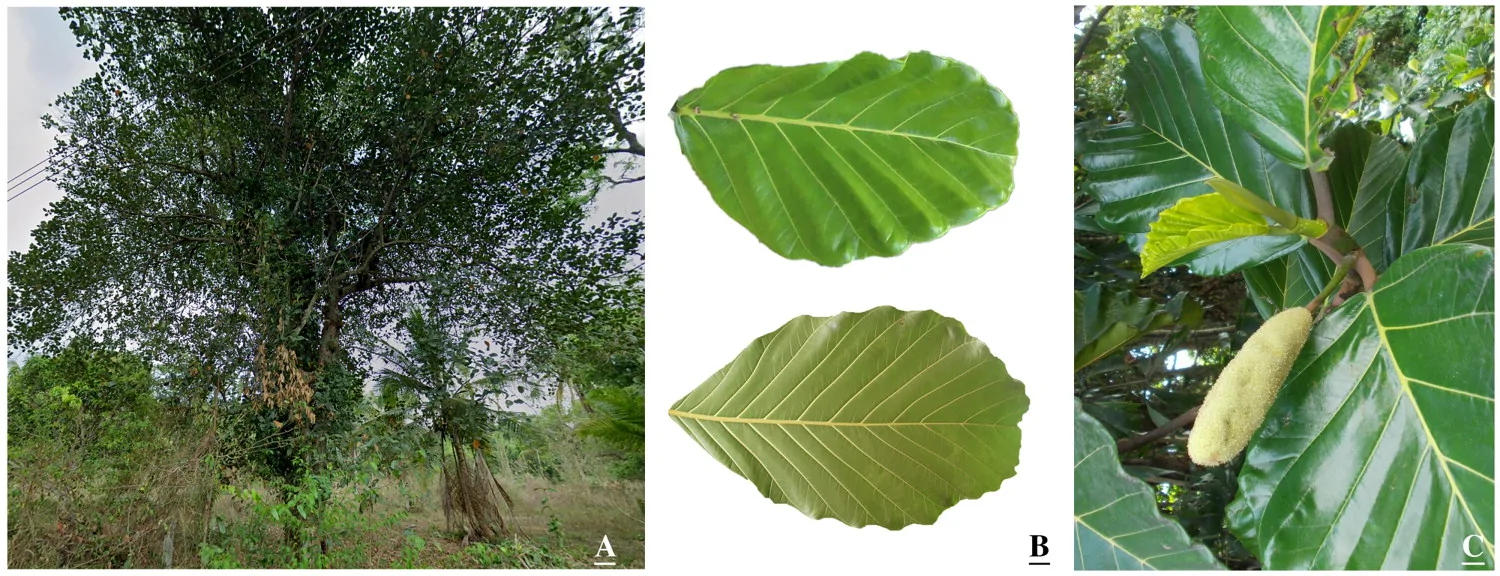
Morphological characteristics of *Artocarpus nobilis*. (A) Mature tree in natural habitat, (B) distinctive leathery long leaves, (C) early stage of a large, round or oval fruit. Photos by N. M. I. Anjalie.

Although studies on *Artocarpus* are abundant due to the importance of several species as staple foods in many regions, genetic and genomic investigations remain comparatively limited, particularly for *A. nobilis*. Based on the NCBI GenBank database, only 21 short gene sequences of *A. nobilis* have been deposited (as of May 14, 2026), comprising four chloroplast gene loci and the nuclear ribosomal internal transcribed spacer (ITS) region. In the wild, *A. nobilis* is threatened by anthropogenic activities, such as deforestation, habitat loss, and overharvesting, resulting in its classification as Vulnerable on the International Union for Conservation of Nature (IUCN) Red List (IUCN, 2026). Therefore, expanding the genomic resources available for this species is essential to support future genetic conservation, population management, and breeding programmes for its sustainable utilization and long-term preservation.

In this study, we sequenced, assembled and annotated the complete chloroplast (cp) genome sequence of *A. nobilis* using next-generation sequencing and bioinformatics tools. Phylogenetic analysis was conducted to evaluate the molecular placement and evolutionary relationship of *A. nobilis* with related species within *Artocarpus*. The genomic information generated in this study will provide valuable molecular resources for evolutionary studies and future conservation efforts within the genus *Artocarpus*

## Materials and methods

Leaf specimens were collected from Udubaddawa, Sri Lanka (7° 28′47.8ʺN 79° 57′12.3ʺE), with voucher specimens deposited at the Herbarium of the University of Jaffna (https://jfn.ac.lk/, Mr. Partheepan Thavarajah, tpartheepan@univ.jfn.ac.lk) under the voucher number UJ/FSC/P/2024-21. No prior approval is required for specimen collection by the local authorities.

Total genomic DNA was extracted using the DNeasy Plant Mini Kit (Qiagen, USA). DNA library preparation was conducted using the TruSeq DNA Sample Prep Kit (Illumina, USA) and next-generation sequencing was carried out on the Illumina NovaSeq platform (Illumina, USA) at Guangzhou Jierui Biotechnology, China. Cp genome assembly was carried out using GetOrganelle v1.7.7.0 (Jin et al. 2020), with the cp genome sequence of *Artocarpus heterophyllus* (GenBank no.: MK303549) as the reference genome. Gene annotation was done using GeSeq v2.03 (Tillich et al. 2017). To avoid misidentification due to local alignment errors in automated annotation pipelines, the annotated *pafI* sequence was extracted and manually BLAST-searched against closely-related cp genomes to validate identity. The genome map and information on the cis- and trans-splicing genes, were generated and identified using CPGview (Liu et al. 2023).

The cp genome sequences of 15 species of *Artocarpus*, with *Broussonetia papyrifera* (GenBank no.: PP584596; Zerega et al. 2010) and *Morus alba* (GenBank no.: OP380683; Clement & Weiblen, 2009) included as outgroup, were used in the phylogenetic analysis. Genome sequence alignment was carried out using MAFFT v7 (Katoh and Standley 2013). The aligned genome sequence dataset was analyzed using both maximum-likelihood (ML) and Bayesian inference (BI) approaches implemented through the RAxML-HPC2 v8.2.12 and MrBayes v3.2.7a pipelines, respectively, available on the CIPRES Science Gateway (Miller et al. 2010). For the ML analysis, the general time-reversible model with gamma-distributed rate variation among sites (GTR+G) was applied, with branch support assessed using 1,000 bootstrap replicates. For the BI analysis, a 4 × 4 nucleotide substitution model together with a mixed nucleotide model were employed. Markov chain Monte Carlo (MCMC) simulations were performed for two million generations, with trees sampled at intervals of every 100 generations. The final tree result was visualized using FigTree v1.4.4 (Rambaut 2018).

## Results

With a minimum coverage of 187× and an average read mapping depth of 1,138 (Supplementary Figure 1), the complete cp genome sequence was 161,196 bp in length, with a GC content of 36% (Figure 2). The large single-copy (LSC) region had a length of 89,878 bp, while the small single-copy (SSC) region was 19,982 bp. The paired inverted repeats (IRs) were each 25,668 bp, separating the LSC and SSC regions (Figure 2). A total of 131 genes were predicted, including 86 CDS, 37 tRNA genes, and eight rRNA genes. Among them, 16 genes contained introns, including 10 CDS (*atpF*, *ndhA*, *ndhB*, *petB*, *petD*, *rpl2*, *rpl16*, *rpoC1*, *rps12*, and *rps16*) and six tRNA genes (*trn*A^-UGC,^ *trn*G^-UCC^, *trn*L^-GAU^, *trn*K^-UUU^, *trn*L^-UAA^, and *trn*V^-UAC^). The *pafI* was validated and confirmed of its identity by comparing its complete gene sequence to at least three published cp genomes, i.e. *A. altilis* (GenBank no: MW367444; De Souza et al. 2021), *A. heterophyllus* (GenBank no: PQ835412; Liu et al. 2017), and *A. tamaran* (GenBank no.: PQ493654; Lestari et al. 2024) *via* BLAST. Two genes, *pafI* and *rps12*, were found to contain two introns. The structure of the trans-splicing gene *rps*12 and the other thirteen cis-splicing genes, including *atpF*, *clpP1*, *ndhA*, *ndhB*, *ndhB*, *pafI*, *petB*, *petD*, *rpl16*, *rpl2*, *rpl2*, *rpoC1*, and *rps16*, were identified (Supplementary Figure 2).

**Figure 2.**
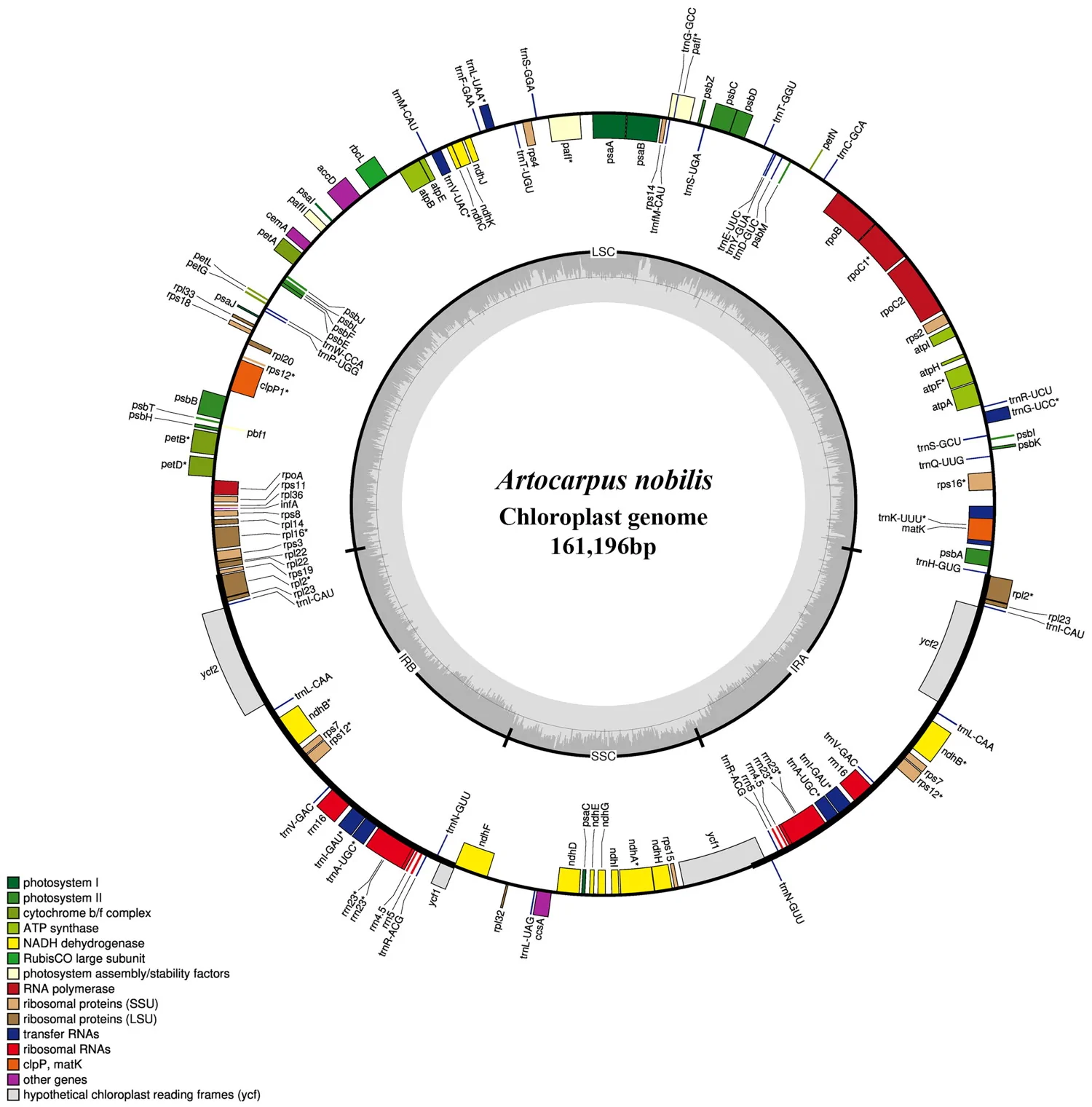
Chloroplast genome map of *Artocarpus nobilis* generated using OGDraw. The first circle represents the annotated genes, with different colors indicating their respective functional categories; the corresponding functional classification is provided in the legend at the bottom left. The second circle delineates the large single-copy, small single-copy, and inverted repeat regions of the plastome. The third circle illustrates the variation in GC content across the genome. Genes on inside of map are transcribed in clockwise direction; genes on outside of map are transcribed in counter clockwise direction.

Both ML and BI analyses produced identical topologies; therefore, only the ML tree is presented, with the posterior probability (PP) value of the BI tree indicated next to the bootstrap support (BS) value (Figure 3). Based on current sampling size, the phylogenetic relationship within *Artocapus* is mostly resolved (BS ≥75%, PP ≥0.95), except for the divergence between *A. nitidus* and *A. petolotii* (PP =0.7). *Artocarpus nobilis* was fully resolved in the present analysis and was closely related to *A. heterophyllus* and *A. integer*.

**Figure 3.**
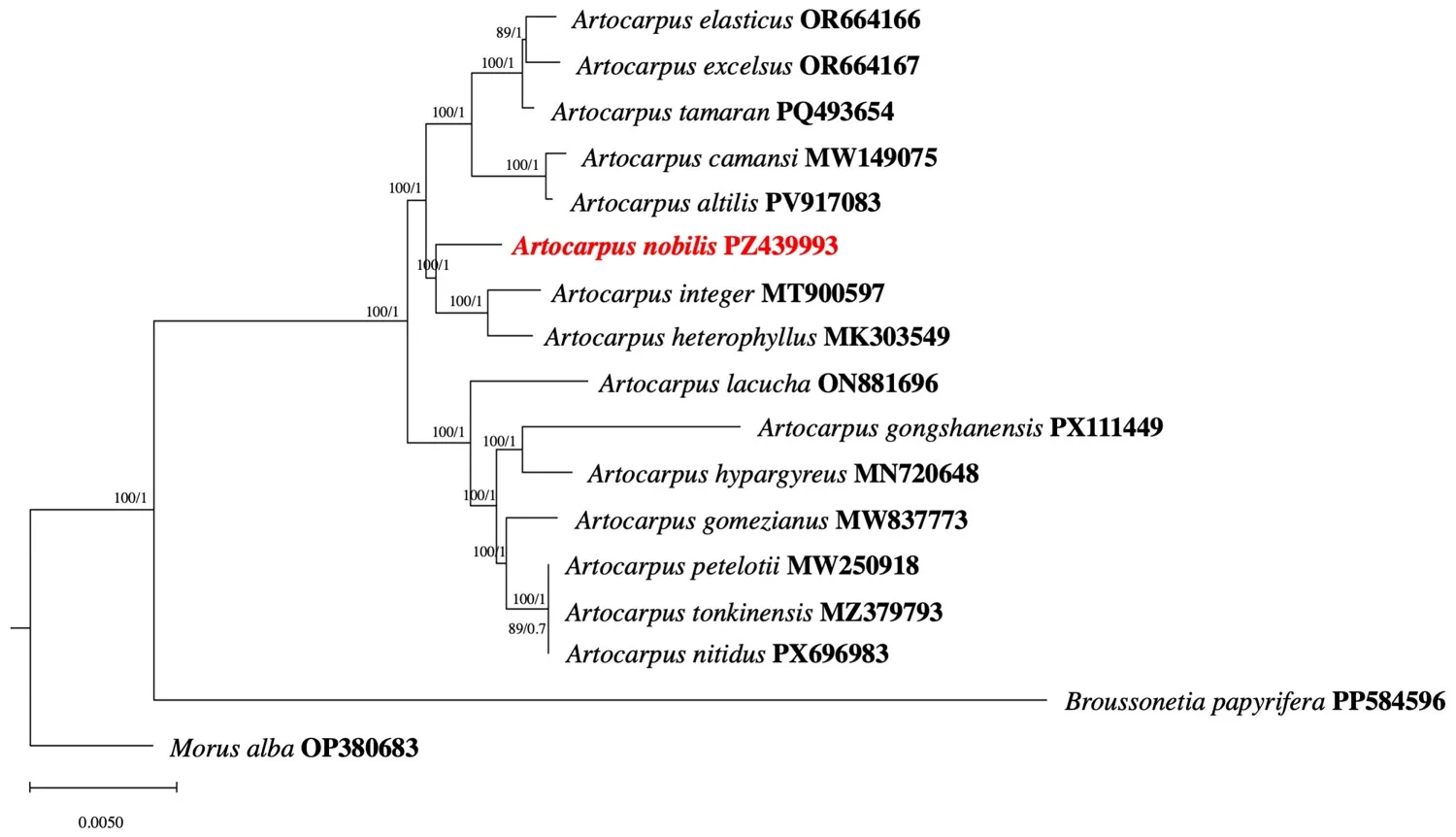
Phylogenetic tree based on the complete chloroplast genome sequence of 13 selected species of *Artocarpus*, with *Broussonetia papyrifera* (GenBank no.: PP584596; Zhou et al. 2025) and *Morus alba* (GenBank no.: OP380683; Zeng et al. 2022) included as outgroup, using PhyloSuite. The bootstrap support (BS; left) and posterior probability (PP; right) values are indicated at each branch node, of which BS ≥ 75% and PP ≥ 0.95 are indicated with an asterisk (*). the following sequences were used: *Artocarpus altilis* (GenBank no.: PV917083; unpublished)*, Artocarpus camansi* (GenBank no.: MW149075; De Souza et al. 2020)*, Artocarpus elasticus* (GenBank no.: OR664166; Qiu et al. 2026)*, Artocarpus excelsus* (GenBank no.: OR664167; unpublished), *Artocarpus gomezianus* (GenBank no.: MW837773; Lin et al. 2021)*, Artocarpus gongshanensis* (GenBank no.: PX111449; unpublished)*, Artocarpus heterophyllus* (GenBank no.: MK303549; Wang et al. 2019)*, Artocarpus hypargyreus* (GenBank no.: MN720648; Li et al. 2020), *Artocarpus integer* (GenBank no.: MT900597; unpublished)*, Artocarpus lacucha* (GenBank no.: ON881696; unpublished)*, Artocarpus nitidus* (GenBank no.: PX696983; Qiu et al. 2026)*, Artocarpus petelotii* (GenBank no.: MW250918; Chen and Liu 2021), *Artocarpus tamaran* (GenBank no.: PQ493654; Lestari et al. 2024)*, Artocarpus tonkinensis* (GenBank no.: MZ379793; Tang et al. 2022)*, Artocarpus nobilis* (GenBank no: PZ439993; this study).

## Discussion

This is the first report on the complete cp genome sequence of *A. nobilis*. The cp genome of *A. nobilis* exhibited a gene structure, size, and gene content were consistent with other published *Artocarpus* cp genomes, i.e. *A. altilis* (Wei et al. 2023), *A. gomezianus* (Lin et al. 2021), and *A. gongshanensis* (Zhang et al. 2026). The low branch support observed in the phylogenetic tree may reflect limited taxon sampling, which can increase phylogenetic error (Magee et al. 2024). Previous studies based on concatenated cp loci and ITS sequences placed *A. nobilis* within section Artocarpus (Senavirathna et al. 2020). Whereas nuclear gene analyses supported its placement in section Duricarpus (Gardner et al. 2020; Gardner and Zerega 2021). In contrast, phylogenetic analysis using complete cp genomes in the present study positioned *A. nobilis* closer to subgenus Cauliflori than to subgenus Artocarpus. This may support recognition of Duricarpus as a distinct subgenus sister to Cauliflori, rather than a section within subgenus Artocarpus. The differing placements likely result from variation in the molecular datasets used, as complete cp genomes may provide evolutionary signal distinct from limited cp loci or nuclear datasets (De Las Rivas et al. 2002). Nevertheless, broader sampling of Duricarpus species is needed to better resolve its phylogenetics relationships.

## Supplementary Material

figs2_2.JPG

figs1_2.JPG

## Data Availability

The genome sequence data that support the findings of this study are openly available in GenBank of NCBI at http://www.ncbi.nlm.nih.gov under the accession number PZ439993. The associated BioProject, SRA, and BioSample numbers are PRJNA1392671, SRR36581763, SAMN54269468 respectively.

## Permission for sample collection

Although *Artocarpus nobilis* is listed as Vulnerable under the IUCN Red List, no permission for sample collection was required by the local authority for research purposes during sampling time.
